# Interpretable machine learning and radiomics in hip MRI diagnostics: comparing ONFH and OA predictions to experts

**DOI:** 10.3389/fimmu.2025.1532248

**Published:** 2025-01-29

**Authors:** Tariq Alkhatatbeh, Ahmad Alkhatatbeh, Qin Guo, Jiechen Chen, Jidong Song, Xingru Qin, Wang Wei

**Affiliations:** ^1^ Comprehensive Orthopedic Surgery Department, the Second Affiliated Hospital of Xi’an Jiaotong University, Xi’an, Shaanxi, China; ^2^ Department of Orthopedics, The First Affiliated Hospital of Shantou University Medical College, Shantou, Guangdong, China; ^3^ Orthopedic Department, the Second Affiliated Hospital of Xi’an Jiaotong University, Xi’an, Shaanxi, China; ^4^ Department of Radiology, the Second Affiliated Hospital of Xi’an Jiaotong University, Xi’an, Shaanxi, China

**Keywords:** radiomics, machine learning, osteonecrosis, osteoarthritis, hip

## Abstract

**Purpose:**

Distinguishing between Osteonecrosis of the femoral head (ONFH) and Osteoarthritis (OA) can be subjective and vary between users with different backgrounds and expertise. This study aimed to construct and evaluate several Radiomics-based machine learning models using MRI to differentiate between those two disorders and compare their efficacies to those of medical experts.

**Methods:**

140 MRI scans were retrospectively collected from the electronic medical records. They were split into training and testing sets in a 7:3 ratio. Handcrafted radiomics features were harvested following the careful manual segmentation of the regions of interest (ROI). After thoroughly selecting these features, various machine learning models have been constructed. The evaluation was carried out using receiver operating characteristic (ROC) curves. Then NaiveBayes (NB) was selected to establish our final Radiomics-model as it performed the best. Three users with different expertise and backgrounds diagnosed and labeled the dataset into either OA or ONFH. Their results have been compared to our Radiomics-model.

**Results:**

The amount of handcrafted radiomics features was 1197 before processing; after the final selection, only 12 key features were retained and used. User 1 had an AUC of 0.632 (95% CI 0.4801-0.7843), User 2 recorded an AUC of 0.565 (95% CI 0.4102-0.7196); while User 3 was on top with an AUC of 0.880 (95% CI 0.7753-0.9843). On the other hand, the Radiomics model attained an AUC of 0.971 (95% CI 0.9298-1.0000); showing greater efficacy than all other users. It also demonstrated a sensitivity of 0.937 and a specificity of 0.885. DCA (Decision Curve Analysis displayed that the radiomics-model had a greater clinical benefit in differentiating OA and ONFH.

**Conclusion:**

We have successfully constructed and evaluated an interpretable radiomics-based machine learning model that could distinguish between OA and ONFH. This method has the ability to aid both junior and senior medical professionals to precisely diagnose and take prompt treatment measures.

## 1 Introduction

Distinguishing osteonecrosis of the femoral head (ONFH) from osteoarthritis (OA) is essential for optimal clinical management, given the significant differences in their underlying pathophysiology, prognosis, and treatment approaches (1, 2). Magnetic resonance imaging (MRI) is fundamental in diagnosis (3), offering excellent soft tissue contrast and the capability to identify early structural changes. Visual interpretation of MRI findings can be subjective and frequently overlaps between osteonecrosis of the femoral head (ONFH) and osteoarthritis (OA), creating challenges for accurate diagnosis, especially in early or end-stage cases (4). A prior study indicated that the diagnoses of osteonecrosis of the femoral head (ONFH) established by radiologists and orthopedic surgeons exhibited only modest concordance. The authors determined the necessity of establishing a consistent, precise, and successful diagnostic technique (5).

Recent advancements in radiomics and machine learning have revolutionized medical imaging analysis, enabling the extraction of high-dimensional quantitative features from MRI data that surpass traditional radiological techniques. Radiomics allows for the quantification of detailed imaging patterns; such as texture, intensity, and shape. Potentially linked to microstructural changes specific to osteonecrosis of the femoral head (ONFH) or osteoarthritis (OA). Studies on knee osteoarthritis demonstrated that MRI-based radiomics could effectively evaluate cartilage (6) and subchondral bone morphology (7), aiding in the diagnosis (8), and prediction of disease severity and progression, including cartilage degeneration (9).

Similarly, radiomics has identified distinct texture and shape characteristics associated with necrotic areas in the hip, allowing early detection and diagnosis of ONFH (10, 11). This approach has also been employed to distinguish between osteosarcoma and chondrosarcoma (12), as well as to differentiate avascular necrosis from transient osteoporosis (13). Furthermore, radiomics has been applied to predict knee pain improvement (14); and valuable insights were given on the use of radiomics in OA through this comprehensive review (15). These developments highlight the growing potential of radiomics to enhance diagnostic accuracy and treatment strategies in musculoskeletal disorders.

This study presents a novel radiomics framework that incorporates SHAP (SHapley Additive Explanations) to distinguish between osteoarthritis (OA) and osteonecrosis of the femoral head (ONFH). SHAP offers a transparent way to interpret machine learning models by breaking down how individual radiomics features contribute to diagnostic predictions. This research is the first to apply such an approach for differentiating OA and ONFH, tackling a critical diagnostic challenge with impressive accuracy.

Unlike earlier studies that primarily focused on knee-related conditions or relied on single- or multi-sequence imaging only to diagnose ONFH, this study evaluates the performance of a radiomics-based model against three health professionals with varying levels of expertise. The results emphasize the model’s consistency and reliability, addressing the variability often seen in human diagnoses and enhancing its potential for real-world clinical use.

By focusing on two disorders that are clinically distinct yet radiologically similar, this study demonstrates how advanced radiomics can refine diagnostic precision and facilitate timely treatment decisions. It has the ability to outperform human experts in certain scenarios while also aiding less experienced physicians in making more accurate diagnoses. Furthermore, the integration of interpretable machine learning ensures that the approach is accessible and practical for everyday use, ultimately improving patient care in different orthopedic settings.

## 2 Materials and methods

### 2.1 Study participants

**Figure 1:**
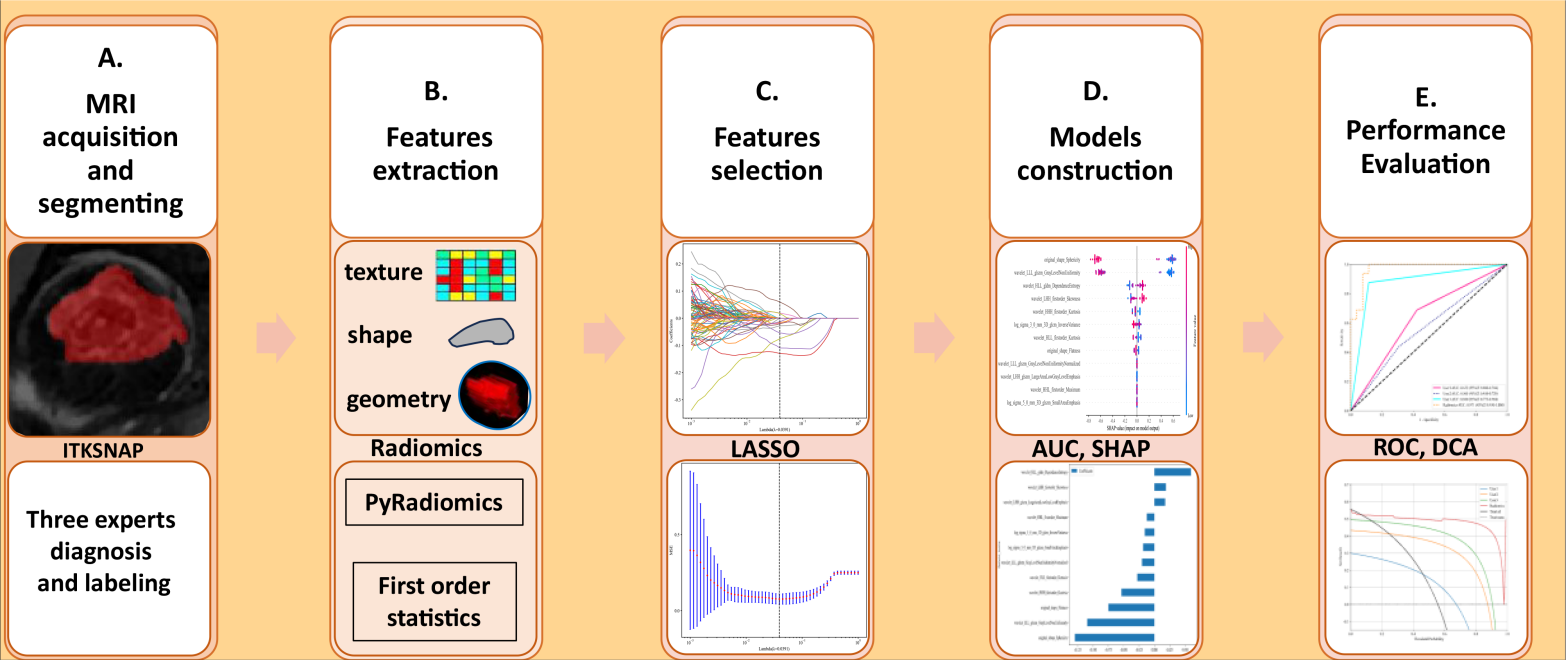
**(A–E)** The methodology and flow of this study, beginning with MRI data gathering and ending with performance evaluation, includes segmentation, feature extraction, selection, and the development of multiple machine learning models. AUC, Area Under the Curve; SHAP, SHapley Additive exPlanations; ROC, Receiver Operating Characteristic; DCA, Decision Curve Analysis.

From February 2016 to April 2024, a senior musculoskeletal radiologist at Xi’an Jiaotong University Second Hospital reviewed radiographs taken by 140 patients; 70 of these patients had osteoarthritis (OA), and 70 had osteoarthritis (ONFH). Thus, the ground truth for our investigation and the development of various machine learning models was based on these. Clinical information and potential risk factors such as a history of steroid usage or alcohol consumption, the presence of a double line sign, and MRI markers of sclerotic and necrotic bone alterations were considered when evaluating MRI results for ONFH patients. The evaluation for OA patients centered on recent indications of inflammation, MRI-confirmed synovitis, elevated subchondral bone signal, bone marrow edema, and joint effusion. Patients were not included in the study if they had any of the following conditions: a history of hip-related problems, collapsing femoral heads, bone cancers, or low-quality MRI pictures. Our hospital’s electronic health record system was the source of the data. See **Figure 1** for a visual representation of the research design pipeline. Patients were not required to provide informed consent for this retrospective analysis, which was approved by our hospital’s ethics review board.

### 2.2 Region of interest segmentation

The 1.5T tesla scanner (Avanto, Siemens Healthineers; Erlangen, Germany) was used to capture the MRI photographs, and the parameters used were: In the coronal plane, in the (headfirst-spine) posture; sequence type turbo spin echo (TSE), T2-weighted with fat suppressed (FS), slice thickness 4.5mm, field of view 640*640mm, acquisition matrix 0\320\240\, echo duration 67ms, repetition time 3000ms.

The ROI on each MRI scan was manually segmented by an orthopedic surgeon with five years of expertise using ITK-SNAP version 4.2.0 (https://www.itksnap.org/). For individuals with osteoarthritis, the region of interest (ROI) included the femoral head and neck. Nevertheless, the ROI was restricted to the necrotic region for patients with ONFH.

### 2.3 Image preprocessing

A 7:3 ratio was used for the random case split between the training and testing groups. In order to train the machine learning models, we used the whole training dataset. To evaluate the models’ performance internally, we used instances from the testing dataset. Unaware of the ground truth diagnosis, three users with varying levels of experience were shown the same set of randomly shuffled radiographs. The first user was an orthopedic surgeon working in our orthopedic department, focusing on sports medicine. A general radiologist who works in the radiology department of the same institution was the second user. The third user, was another orthopedic surgeon from an external hospital. He is focused on OA, ONFH, and arthroplasty operations. We handled any differences in voxel spacing in this experiment using the fixed-resolution resampling approach. By resampling each image to a 1*1*1 mm size, the voxel spacing was uniform across all images. The data was finally normalized using z-scores, another name for zero-mean normalization.

### 2.4 Features extraction

This study’s feature extraction used traditional, hand-crafted radiomic features—geometry, intensity, and texture—derived from the initial radiographs. In order to extract radiomic characteristics, PyRadiomics was employed. The geometric, intensity and textural types of manually generated radiomic features are the three main groups. The geometric features relate to the three-dimensional shape of the bone cells. An analysis of the statistical distribution of voxel intensities within the femoral head is performed by the intensity features using first-order statistics. Features that characterize patterns or spatial distributions of intensities beyond the first order are indicated by the texture features. Texture features are retrieved using a variety of methodologies, such as the gray-level run length matrix (GLRLM), neighborhood gray-tone difference matrix (NGTDM), gray-level size zone matrix (GLSZM), and the gray-level co-occurrence matrix (GLCM). The nonlinear intensity of picture voxels is transformed using a number of transformations—including Square, Square Root, Logarithm, Gradient, LBP3D, and Exponential—in order to attain high-throughput features. One, two, and three are the sigma values used by the high Laplace filter. In addition, eight wavelet transform algorithms—HLL, HLH, HHL, LHH, LLL, LLH, and LHL—were used in the process of obtaining first-order statistics and texture features. The web resource at https://pyradiomics.readthedocs.io/en/latest/features.html provides a thorough explanation of all radiography features.

### 2.5 Features selection

The features were first standardized using the z-score standardization method approach before going through three screening stages before final selection. First, all features were subjected to the Mann-Whitney U test; features with a P-value below 0.05 were kept. Then, to identify highly correlated features, the Pearson test was used. In order to be considered potentially predictive, features needed a P-value lower than 0.05. Finally, the least absolute shrinkage and selection operators (LASSO) were used to evaluate the key features in the end.

### 2.6 Radiomics models construction

After LASSO was used to identify the key features, we passed them into various machine learning classifiers such as XGBoost, NaiveBayes (NB), Random Forests (RF), Logistic Regression (LR), K-Nearest Neighbors (KNN), Support Vector Machines (SVM), and others. We selected the best performer after comparing all of them to construct the final model. We employed 5-fold cross-validation in this particular instance.

### 2.7 Statistical analysis

We used the Python Statsmodels package (0.13.2 version) to evaluate the data, and we deemed a p-value below 0.05 statistically significant. Using ROC curves and the associated diagnostic accuracy, sensitivity, specificity, positive predictive value (PPV), and negative predictive value (NPV), we assessed the clinical significance of the models in distinguishing between ONFH and OA. Decision curve analysis (DCA) and calibration curves were also used to evaluate the discriminative capacity of the model. To further evaluate the model’s robustness, we employed the Hosmer-Lemeshow test.

## 3 Results

### 3.1 Patients’ characteristics

The study comprised a total of 140 patients’ MRI scans, consisting of 70 OA individuals and 70 ONFH patients. The patients were categorized into a training group of 98 individuals and a testing group of 42 individuals. **Table 1** demonstrates a summary of the patient’s primary attributes.

**Table 1:** These are the fundamental characteristics of a total of 140 patients.

Characteristic	ONFH patients (n=70)	OA patients (n=70)
**Age (years)** **Mean ± SD**	47.67 ± 15.387	59.16 ± 12.605
Gender, No. (%)		
**Male**	34 (48.6%)	25 (35.7%)
**Female**	36 (51.4%)	45 (64.3%)

SD, standard deviation.

### 3.2 Feature extraction and selection

A grand total of 1,119 Radiomics features were extracted using a tool that is specifically designed for feature analysis and is part of Pyradiomics (http://pyradiomics.readthedocs.io). There was a total of 234 First Order features, 182 GLRM features, 208 GLSLSM features, 65 NGTDM features, 286 GLCM features, and 14 shape features.

For each of the selected features, we ran a feature screening and a Mann-Whitney U test. The final count was 1006 features, with features being maintained only if their P-value was less than 0.05.

The second step was to use the Pearson correlation coefficient, a measure of feature correlation, to assess features with good repeatability. When two features had a correlation coefficient greater than 0.9, just single one of them was retained. In the end, 215 features were retained.

Step three involved using the logistic regression model (LASSO) to narrow down the feature set for the model’s construction and decrease the number of features overall. All regression coefficients are shrunk by LASSO until they approach zero, and the coefficients of insignificant features are adjusted to zero according to the regulation weight Lambda (λ). The optimal value of λ was found by performing a 10-fold cross-validation with a minimum criteria method. The final value of λ was chosen based on its ability to produce the lowest cross-validation error. After integrating the features with non-zero coefficients, a Radiomics model was built using the features used to construct a regression model. Following this, we determined a patient’s radiomics score by adding all the retained features multiplied by their corresponding model coefficients.

**Figure 2:**
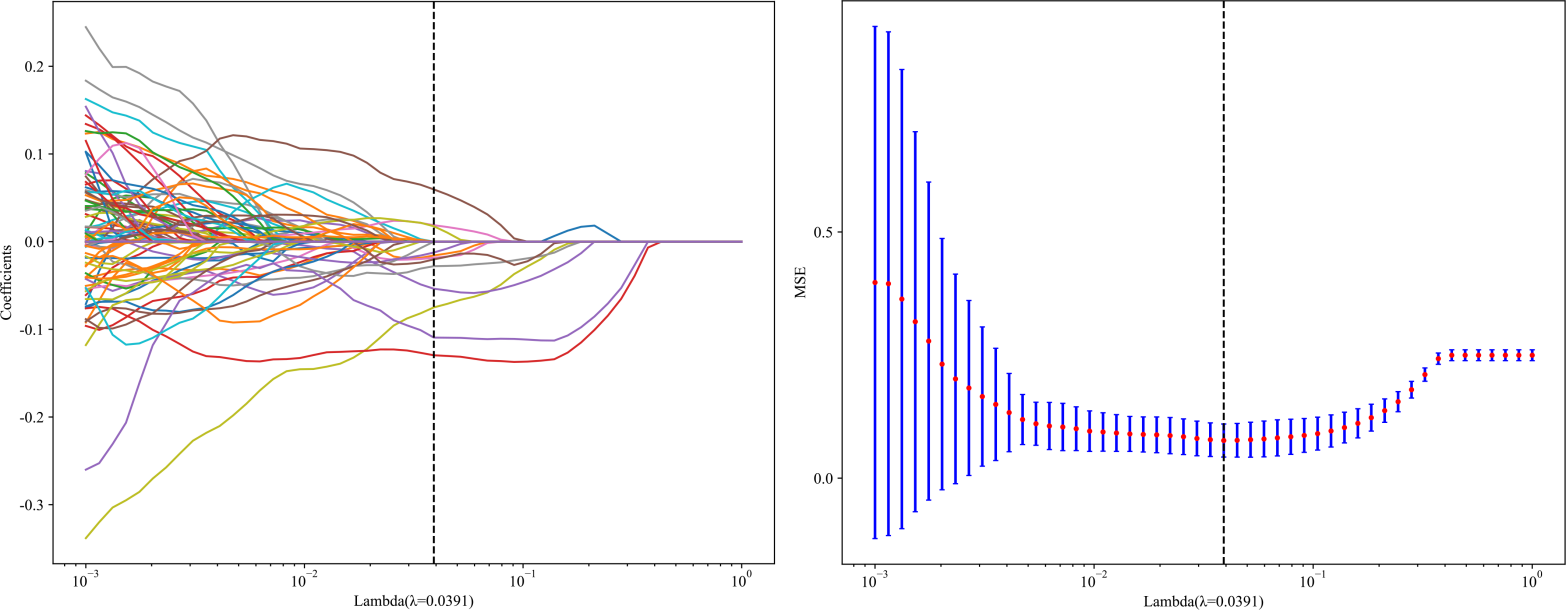
LASSO Coefficients profile plot with various log (λ) is displayed (left); the vertical dashed line represents the selected features with nonzero coefficients chosen to the optimal lambda. (right) MSE of 10-fold cross-validation for the most valuable features screened for the Radiomics-model.

**Figure 3:**
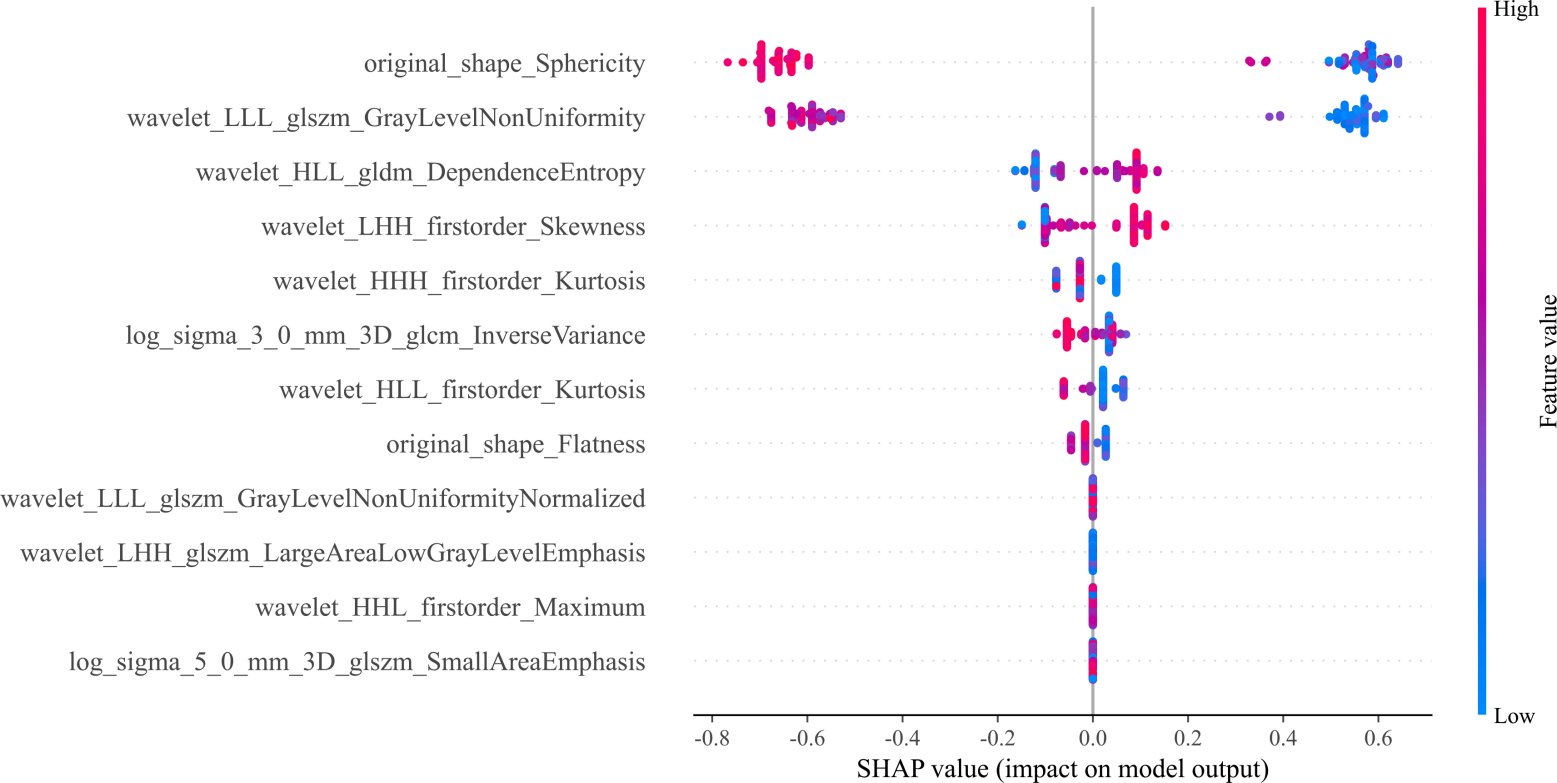
The 12 most important features are displayed in the bee swarm plot of SHAP values, in order of importance. The SHAP values quantify the contribution of each factor to the prediction outcome; higher values indicate greater influence. The horizontal axis displays this information. Each feature’s ranking is based on its relative weight in making the prediction. Here, we can see the distribution of feature values along the vertical axis, which is colored on a gradient from low (blue) to high (red). A feature’s impact at a given level is proportional to the density of points falling within that level’s SHAP value range.

**Figure 4:**
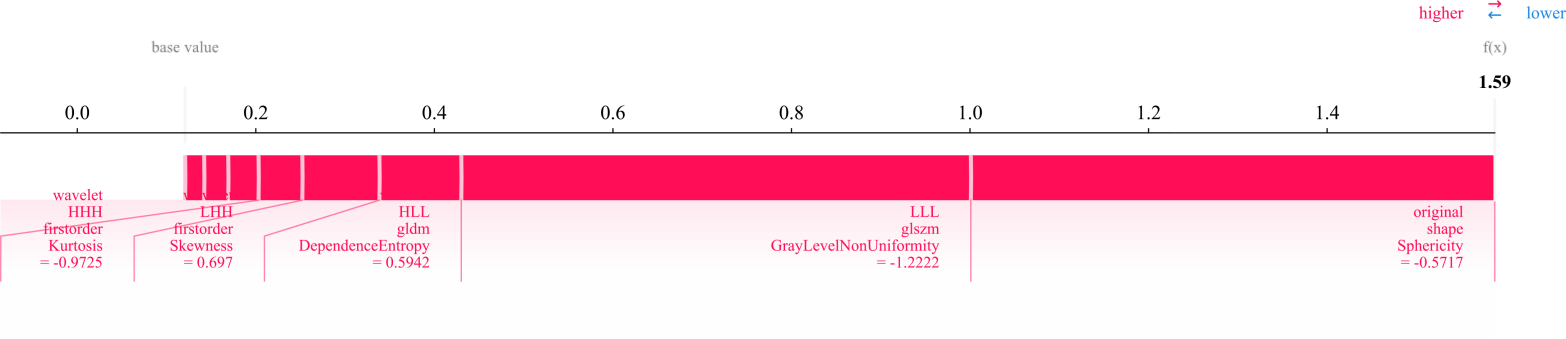
SHAP force plot illustrating the contribution of 12 radiomic features in differentiating osteonecrosis of the femoral head (ONFH) from osteoarthritis (OA) based on MRI. The base value represents the model’s average prediction, with the final prediction (f(x) = 1.59) derived from the cumulative feature contributions. Red bars indicate the direction and magnitude of each feature’s influence, with key metrics capturing intensity distribution, texture complexity, and geometric shape (e.g., sphericity).

Twelve radiomics-related features were identified by LASSO regression modeling, which was executed using the scikit-learn package in Python. Below, you can see a plot of the LASSO models’ coefficient profiles and the mean square errors (MSE) that were derived from 10-fold validation. Every independent predictor’s changing trajectory is shown by each curve in the plot. **Figure 2** (left) Describes how LASSO logistic regression is used to choose features in the Radiomics model. **Figure 2** (right) Displays the mean squared error (MSE) values derived from a 10-fold cross-validation of the Radiomics model. For the purpose of interpreting and visualizing the radiomic features that were utilized in the radiomics models, the SHAP (16) approach was utilized in **Figure 3**. This technique draws attention to the significance of individual features within the context of a complicated machine learning framework. It provides insights into how each feature contributes to the probability of a particular outcome. Moreover, the SHAP force plot in **Figure 4** summarizes how each selected radiomic feature shifts the model’s prediction from the baseline (average) output toward the final predicted value. This means it displays the most influential features in classifying the given MRI case (Case 1 in our dataset) as either OA or ONFH. The length and direction of each red bar indicate how much that particular feature “pushes” the model’s decision. Positive SHAP values suggest that the feature’s measured value for this particular case pushes the model’s prediction toward the chosen positive class (e.g., ONFH), Whereas negative SHAP values indicate that the feature’s value nudges the model’s prediction away from the positive class, potentially supporting the alternative classification (e.g., OA).

### 3.3 Predictive performance of the radiomics-model

**Figure 5:**
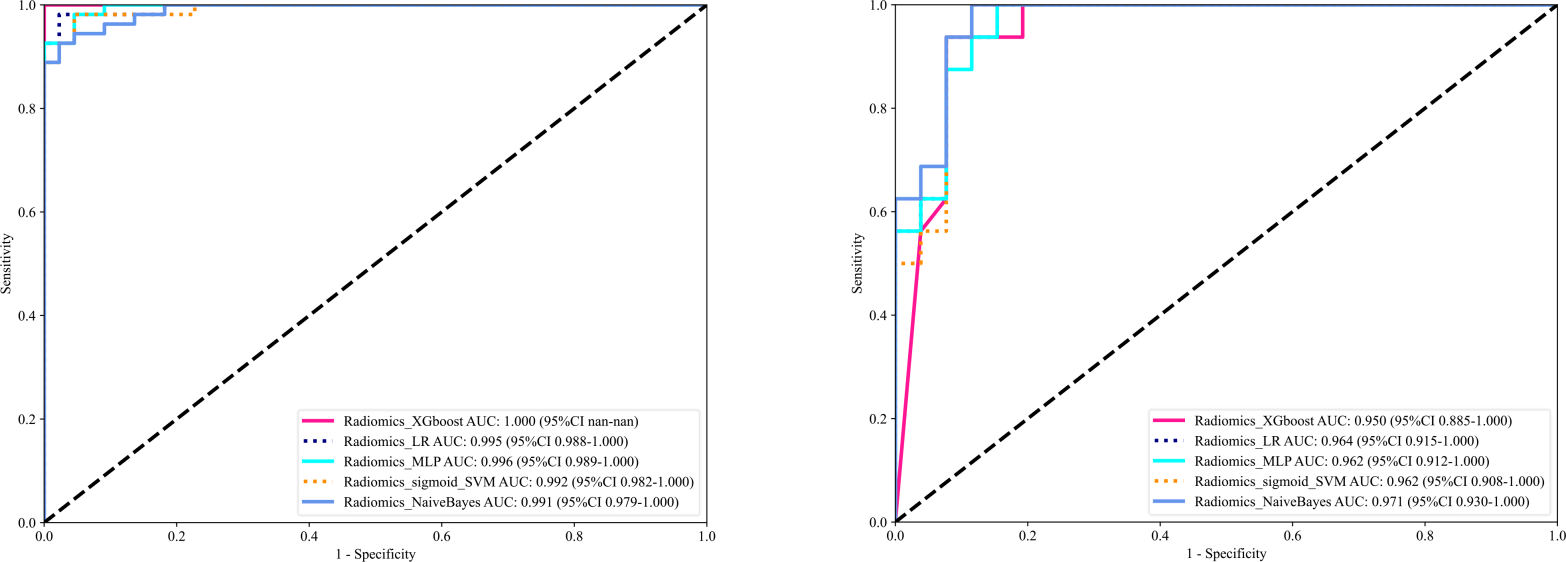
Shows the ROC curves of different machine learning models ROC curves in the training cohort (left), and the testing cohort (right).

XGBoost (17), LR (18), MLP (19), sigmoid_SVM (20), and NB (21) based models were constructed and compared; their AUCs in the testing cohort were (0.950, 0.964, 0.962, 0.962, and 0.971) respectively as shown in **Figure 5**. As NB performed the best, it was the classifier of choice for in constructing the final Radiomics-model. On the other hand, the performance of User 1(AUC=0.606) and (AUC=0.632); User 2 (AUC=0.819) and (AUC=0.565); User 3 (AUC=0.886) and (AUC=0.880) in the training and testing cohorts respectively.

**Figure 6:**
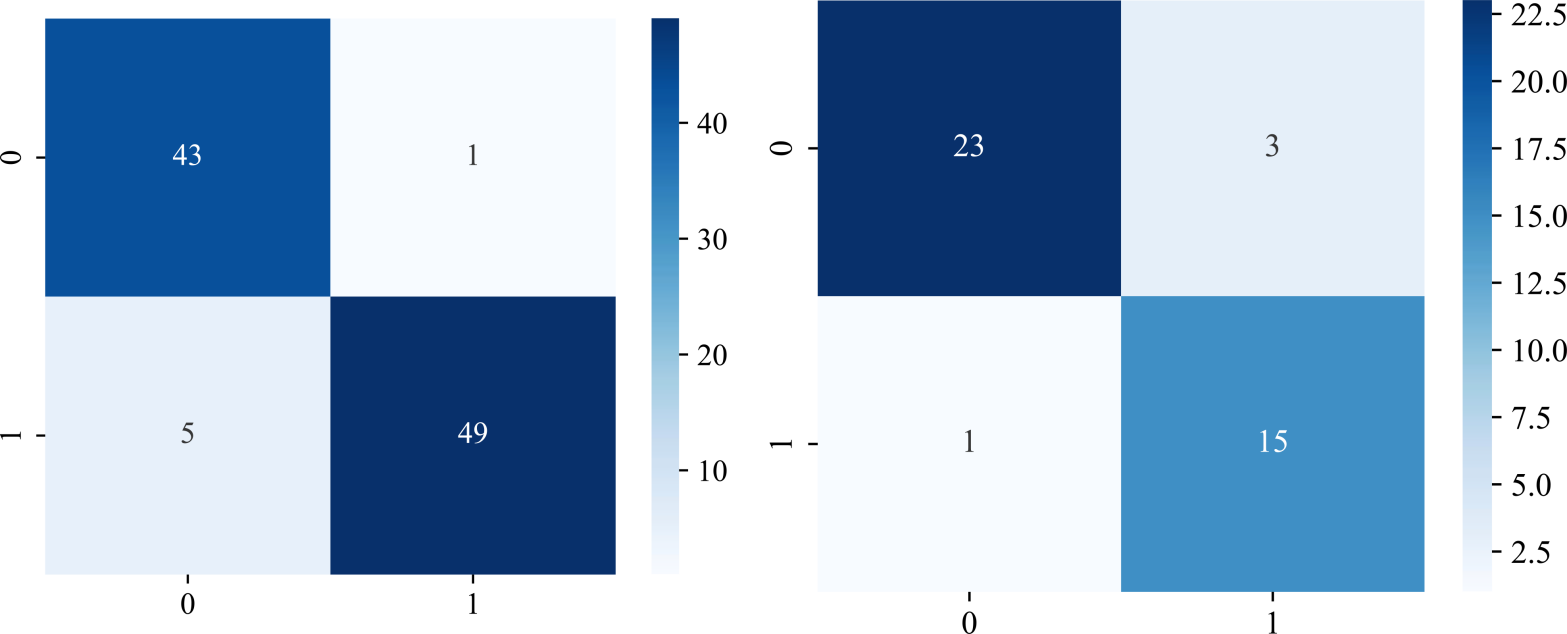
Evaluation of the Radiomics model using confusion matrices. The performance of the model is presented in both the training cohort (left), and the testing cohort (right). Class labels are designated as “OA” (0) and “ONFH” (1). The values are as follows: (TP) = 43 and 23; (TN)= 49 and 15; (FP)= 1 and 3; (FN)= 5 and 1 in the training and testing cohorts respectively.

**Figure 7:**
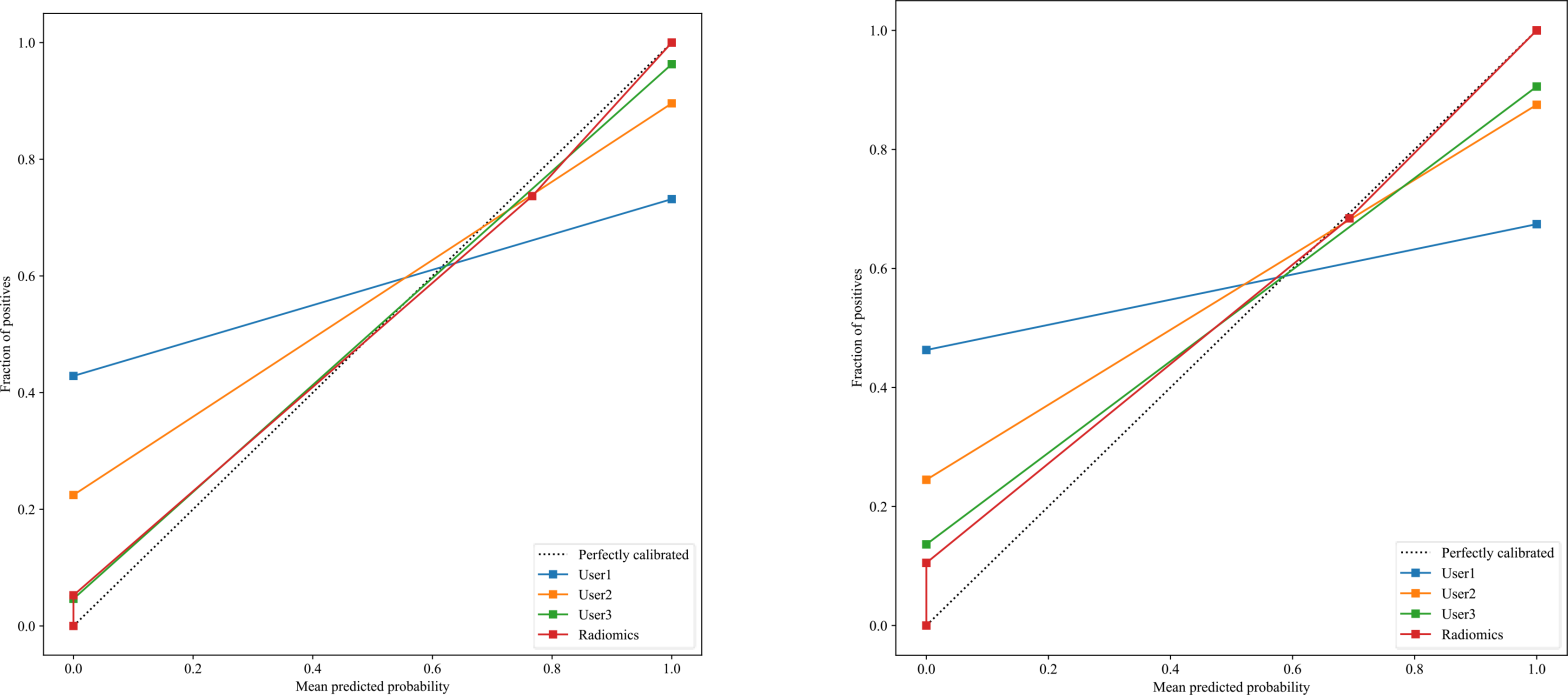
There is a significant association between the average predicted probability (x-axis) and the proportion of positive outcomes (y-axis) in the calibration curves for the Radiomics-model in the testing group (right) and the training group (left), showing that calibration was successful with the perfectly calibrated line.

**Figure 8:**
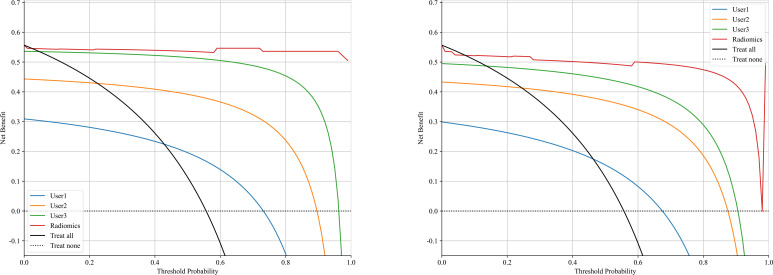
Shows a decision curve analysis was performed on the Radiomics-model across the training group (left) and the testing group (right). The y-axis represents the net benefit, while the x-axis represents the threshold probability.

The significant features selected for the Radiomics-model are presented in **Table 2**. The diagnostic AUC, 95%CI, accuracy, sensitivity, specificity, PPV, and NPV of all three users and the Radiomics-model are demonstrated in **Table 3**. The confusion matrix for the Radiomics model is presented in **Figure 6**, comprising True Positives (TP), True Negatives (TN), False Positives (FP), and False Negatives (FN). In addition, the calibration curves showed good agreement between the Radiomics based model and the perfectly calibrated line, as shown in **Figure 7**. The P-values of the Hosmer-LemeShow test in **Table 4** were 0.574, and 0.329 for the Radiomics model in the training and testing cohort respectively. This indicates a good-fitting model, as all of the values were greater than 0.05. Both the CLEAR (22) and METRICS (23) checklists of this study were presented in **Supplementary Data sheet 1** and **Supplementary Data sheet 2**. Furthermore, the radiomic signatures extracted from each tested patient have been provided in **Supplementary Data sheet 3**. Besides, the net benefit was plotted against threshold probability in **Figure 8**, which displays the Decision curve analysis (DCA); it designates that the Radiomics-model has the highest net benefit in differentiating between OA and ONFH. **Supplementary Figures 10** and **11** present heatmaps that display the radiomics features selected in the study.

**Table 2:** Displays the key radiomics characteristics chosen using LASSO analysis.

Sequence	Name
	‘log_sigma_3_0_mm_3D_glcm_InverseVariance’, ‘log_sigma_5_0_mm_3D_glszm_SmallAreaEmphasis’, ‘original_shape_Flatness’, ‘original_shape_Sphericity’, ‘wavelet_HHH_firstorder_Kurtosis’, ‘wavelet_HHL_firstorder_Maximum’, ‘wavelet_HLL_firstorder_Kurtosis’, ‘wavelet_HLL_gldm_DependenceEntropy’, ‘wavelet_LHH_firstorder_Skewness’, ‘wavelet_LHH_glszm_LargeAreaLowGrayLevelEmphasis’, ‘wavelet_LLL_glszm_GrayLevelNonUniformity’, ‘wavelet_LLL_glszm_GrayLevelNonUniformityNormalized’

**Table 3:** All the metrics for User 1, 2, 3, and the NB-model.

Model	Cohort	AUC	AUC 95% CI	ACC	Acc 95% CI	SEN	SPE	PPV	NPV
User 1	Train	0.606	0.5081 - 0.7033	0.443	0.3424 - 0.5477	0.000	1.000	0.000	0.443
User 2	Train	0.819	0.7425 - 0.8958	0.443	0.3424 - 0.5477	0.000	1.000	0.000	0.443
User 3	Train	0.886	0.8220 - 0.9506	0.443	0.3424 - 0.5477	0.000	1.000	0.000	0.443
NB-model	Train	0.991	0.9790 - 1.0000	0.938	0.8702 - 0.9770	0.907	0.977	0.980	0.894
User 1	Test	0.632	0.4801 - 0.7843	0.619	0.4564 - 0.7643	0.000	1.000	0.000	0.619
User 2	Test	0.565	0.4102 - 0.7196	0.619	0.4564 - 0.7643	0.000	1.000	0.000	0.619
User 3	Test	0.880	0.7753 - 0.9843	0.619	0.4564 - 0.7643	0.000	1.000	0.000	0.619
NB-model	Test	0.971	0.9298 - 1.0000	0.905	0.7738 - 0.9734	0.937	0.885	0.833	0.958

AUC, Area under the curve; ACC, accuracy; SEN, Sensitivity; SPE, specificity; PPV, positive predictive value; NPV, negative predictive value.

**Table 4:** Illustrates the significance levels (P values) obtained by the Hosmer-Lemeshow test, which is used to assess the goodness of fit of models.

Model	NB-model	User 1	User 2	User 3	Cohort
**P**	0.574	0.321	0.336	0.240	Train
**P**	0.329	0.078	0.171	0.133	Test

P, P-value.

## 4 Discussion

**Figure 9:**
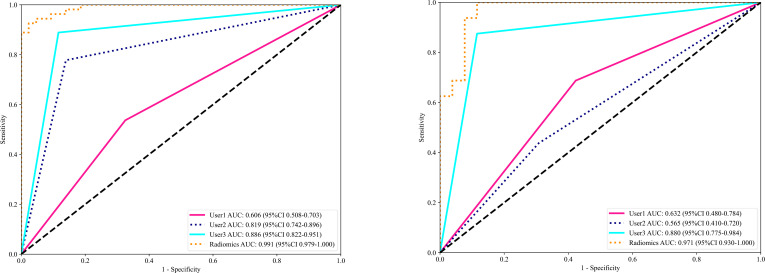
The receiver operating characteristic (ROC) of the Radiomics model and all three users in the training set (left) and the testing set (right) are displayed. The findings for the radiomics model are superior to those of other users, with an area under the curve (AUC) of 0.971.

In this study, we presented how Radiomics can be utilized to make a machine learning-based Radiomics model that can differentiate between OA and ONFH accurately; AUC= 0.968 (95%CI 0.909-1.000). This model has shown superior results to three different users with various expertise levels, User 1 (AUC=0.944 (95%CI 0.862-1.000)) and User 2 (AUC=0.930 (95%CI 0.838-1.000)) and User 3 (AUC=0.880(95%CI 0.7753 - 0.9843)). The ROC for the Radiomics-model with three users is illustrated in **Figure 9**. Our study was based on a single sequence MRI (T2W1), which gives a great value to our model, as differentiating between early or end-stage OA and ONFH solely depending on a single sequence MRI regardless of the clinical symptoms and a thorough background checking can be challenging even for experienced surgeons. But our model was able to accurately achieve this task effectively.

Wei Li used radiomics based on plain radiographs to grade knee OA severity (24); his study population consisted of 475 patients, and X-ray images were processed using a radiomic feature selection and dimensionality reduction technique. He found that combining information from anteroposterior and lateral images significantly improved the model’s performance for diagnosing knee osteoarthritis. Their model outperformed radiologists in diagnosing knee OA. Francesca, by combining CT and MRI data and employing radiomics, aimed to predict knee cartilage degeneration in OA (9). Her findings indicated the potential of radiomics as a promising tool in clinical practice for early diagnosis and assessment of cartilage degeneration. Li presented a comprehensive study on utilizing radiomics signatures and age as a nomogram model for diagnosing knee osteoarthritis (25). Shengfa presented a method using Radiomics and Neural network for knee OA incidence prediction by integrating meniscus and cartilage features (26). Kaibin investigated the use of radiomics for the analysis of hip CT to screen osteoporosis (27). Other papers provided valuable insights into radiomics applications in orthopedics but differ significantly from the current work in focus (28). developed a radiomics-based decision support tool for cervical disc degeneration grading using combined T1 and T2 MRI modalities, achieving an AUC of 0.95. Their study emphasized the value of multi-modality integration and higher-order texture features but focused on cervical spine pathology rather than conditions involving the femoral head (10). utilized multi-sequence MRI (T1, T2, and Cor STIR) to diagnose early osteonecrosis of the femoral head (ONFH), achieving an AUC of 0.94, and highlighted the benefits of combining multiple sequences to enhance diagnostic performance. Similarly (11), used a single-sequence MRI-based deep learning radiomics model for ONFH diagnosis, achieving an AUC of 0.968 but with limited focus on model interpretability. **Table 5** compares the previously published papers relative to this topic.

**Table 5:** The table provides a comparison of key studies on radiomics applications in musculoskeletal imaging.

Study	Objective	Sample Size & Imaging Modality	Key Features/Techniques	Performance Metrics	Primary Findings
**Klontzas et al., 2021 (** 13)	Differentiate transient osteoporosis (TOH) from avascular necrosis (AVN) using MRI radiomics and machine learning.	213 hips (109 TOH, 104 AVN); MRI	XGBoost, CatBoost, and SVM classifiers with 38 radiomics features.	AUC: 0.937 (XGBoost).	Radiomics-based ML achieved similar performance to musculoskeletal radiologists and significantly outperformed general radiologists.
**Xue et al., 2022 (** 7)	Use MRI-based radiomics for subchondral bone analysis to identify knee OA.	88 knees (56 with OA); MRI (3T, sagittal 3D BFFE sequence).	LASSO-selected radiomics features; SVM model constructed.	AUC: 0.961 (radiomics)	MRI-based radiomics outperformed traditional structural parameter analysis for OA classification.
**Li et al., 2023 (** 25)	Create a nomogram model combining radiomics signatures and age to diagnose knee OA.	4403 knee X-rays from 1174 patients.	Radiomics feature selection using LASSO; Logistic regression (LR) model developed.	AUC: 0.847 (nomogram), 0.843 (radiomics model).	Combining radiomics with clinical data (age) enhances diagnostic accuracy and clinical utility.
**Cui et al., 2023 (** 29)	Develop machine learning models for MRI-based radiomics to diagnose knee OA.	148 patients (117 training, 31 validation).	LASSO for feature selection; Logistic Regression, KNN, and SVM models evaluated.	AUC: 0.984 (training), 0.983 (validation).	MRI radiomics showed excellent performance in non-invasive and preoperative OA diagnosis.
**Li et al., 2024 (** 8)	Develop a bone marrow edema model using MRI-based radiomics to diagnose knee osteoarthritis (OA).	302 patients (211 training, 91 testing); MRI	Extracted 11 radiomics features from bone marrow edema; Logistic regression and nomogram developed combining clinical characteristics.	AUC: 0.906 (training), 0.845 (testing).	MRI-based radiomics combined with clinical features demonstrated superior diagnostic performance compared to clinical models alone.
**Angelone et al., 2024 (** 9)	Explore radiomics and machine learning to predict knee cartilage degeneration in OA.	138 knees (MRI and CT scans)	Texture and shape-related radiomics features; Machine learning algorithms for classification.	Accuracy SVM Linear = 90.25 (±7.03)	Radiomics demonstrated potential for early OA detection and personalized treatment.
**Gao et al., 2024 (** 12)	Develop radiomics models to differentiate osteosarcoma (OS) and chondrosarcoma (CS) using MRI.	87 training, 29 validation; MRI (CET1 and T2WI-FS sequences).	LASSO-selected features; Multivariate logistic regression.	AUC: 0.970 (training, T2WI-FS), 0.899 (validation).	Radiomics models effectively differentiated OS and CS with high accuracy and diagnostic value.
**Li et al., 2024 (** 24)	Construct a radiomics-based automatic grading model for knee OA using plain radiographs.	473 knee joints (AP and LAT radiographs).	Radiomics feature selection; Logistic regression for classification.	AUC: 0.727 (combined AP & LAT images).	Combining radiographic views enhances radiomics model performance for OA grading.
**Wang et al., 2024 (** 10)	Develop a multi-sequence MRI-based radiomics model for early osteonecrosis of the femoral head (ONFH).	244 total (122 ONFH, 122 normal); Multi-sequence MRI.	LASSO and mRMR feature selection; Multi-sequence radiomics model.	AUC: 0.94 (test set).	Multi-sequence radiomics model outperformed radiologists in early ONFH diagnosis.
**Alkhatatbeh et al., 2024 (** 11)	Develop a deep learning-based radiomics model for early ONFH using single-sequence MRI.	150 patients (80 healthy, 70 necrotic).	Logistic regression; Combined handcrafted and deep learning features.	AUC: 0.968 (DLR model).	Single-sequence MRI with deep learning-based radiomics provided high diagnostic accuracy for early ONFH.
**Xie et al., 2024 (** 28)	Develop and validate an MRI radiomics-based decision support tool for automated grading of cervical disc degeneration.	2,610 cervical disc samples from 435 patients (T1 & T2 MRI).	mRMR for feature selection; Random Forest for modeling; Combined radiomics model using T1 and T2 MRI modalities.	AUC: 0.95 (test set).	The decision support tool demonstrated robust diagnostic performance for cervical disc degeneration and facilitated individualized management.
**Current Study**	Develop a radiomics model to differentiate between ONFH and OA using MRI and machine learning techniques.	140 patients (70 ONFH, 70 OA); Single-phase MRI.	Radiomics features selected using LASSO; SHAP analysis used for feature importance; NaiveBayes as a classifier.	AUC: 0.955 (test set).	The proposed model demonstrated high diagnostic accuracy and robustness in distinguishing ONFH from OA, surpassing three healthcare professionals.

"Imaging Modality" indicates the type of MRI sequences or modalities used in each study. "Target Condition(s)" lists the specific diseases or conditions addressed. "Key Radiomics Features/Models" highlights the primary features or machine learning models utilized. "Sample Size" indicates the number of participants or samples included in the analysis. "Performance Metrics" report the diagnostic accuracy, typically measured by AUC (Area Under the Curve). "Key Findings and Contributions" summarize the study’s major outcomes and its impact on the field.

Bold text represents the authors names for each of the studies with the corresponding citation.

The present study uniquely addresses the critical clinical challenge of distinguishing between osteoarthritis (OA) and osteonecrosis of the femoral head (ONFH)—two conditions with overlapping imaging characteristics but requiring distinct treatment strategies. By employing a SHAP-interpreted radiomics framework and utilizing a single-phase MRI, this work balances simplicity and accuracy while prioritizing feature interpretability, offering a robust and clinically applicable tool to improve diagnostic precision and support timely intervention. This paper represents the first investigation into the application of radiomics for differentiating between hip OA and ONFH. While most research focuses on the knee as it is the primary site of osteoarthritis, hip osteoarthritis ranks second in prevalence, highlighting the need for further study in this area. The interpretability of the presented model emphasizes the significance of individual radiomic features within a complex machine learning framework and provides insights into their contribution to specific diagnostic outcomes.Our study has certain limitations, including a moderate sample size for both training and testing. In addition,the investigation was conducted at a single center only; a multi-center study in the future could provide a more comprehensive examination of Radiomics in distinguishing between OA and ONFH. Moreover, involving more senior specialists could enhance the usefulness of this study. In conclusion, our research developed a Radiomics Model utilizing radiomics and machine learning to differentiate between OA and OFH. This strategy surpassed three users with varying levels of expertise. This innovative approach can provide critical diagnostic information and improve early treatment planning for patients with either osteoarthritis or osteonecrosis of the femoral head.

## 5 Conclusion

A Machine Learning-Based Radiomics model was developed and evaluated, demonstrating effective differentiation between ONFH and OA. This model has the potential to benefit both junior and senior surgeons, as well as radiologists, by facilitating early accurate diagnosis and the development of timely treatment plans.

## Data Availability

The raw data supporting the conclusions of this article will be made available by the authors, without undue reservation.
