# Supplementary material for: Interpretable machine learning and radiomics in hip MRI diagnostics: comparing ONFH and OA predictions to experts
Source: Front Immunol. 2025 Jan 29;16:1532248. doi: 10.3389/fimmu.2025.1532248 (PMC11813894; doi:10.3389/fimmu.2025.1532248)
Supplement: Supplementary file 4 [file DataSheet4.docx]

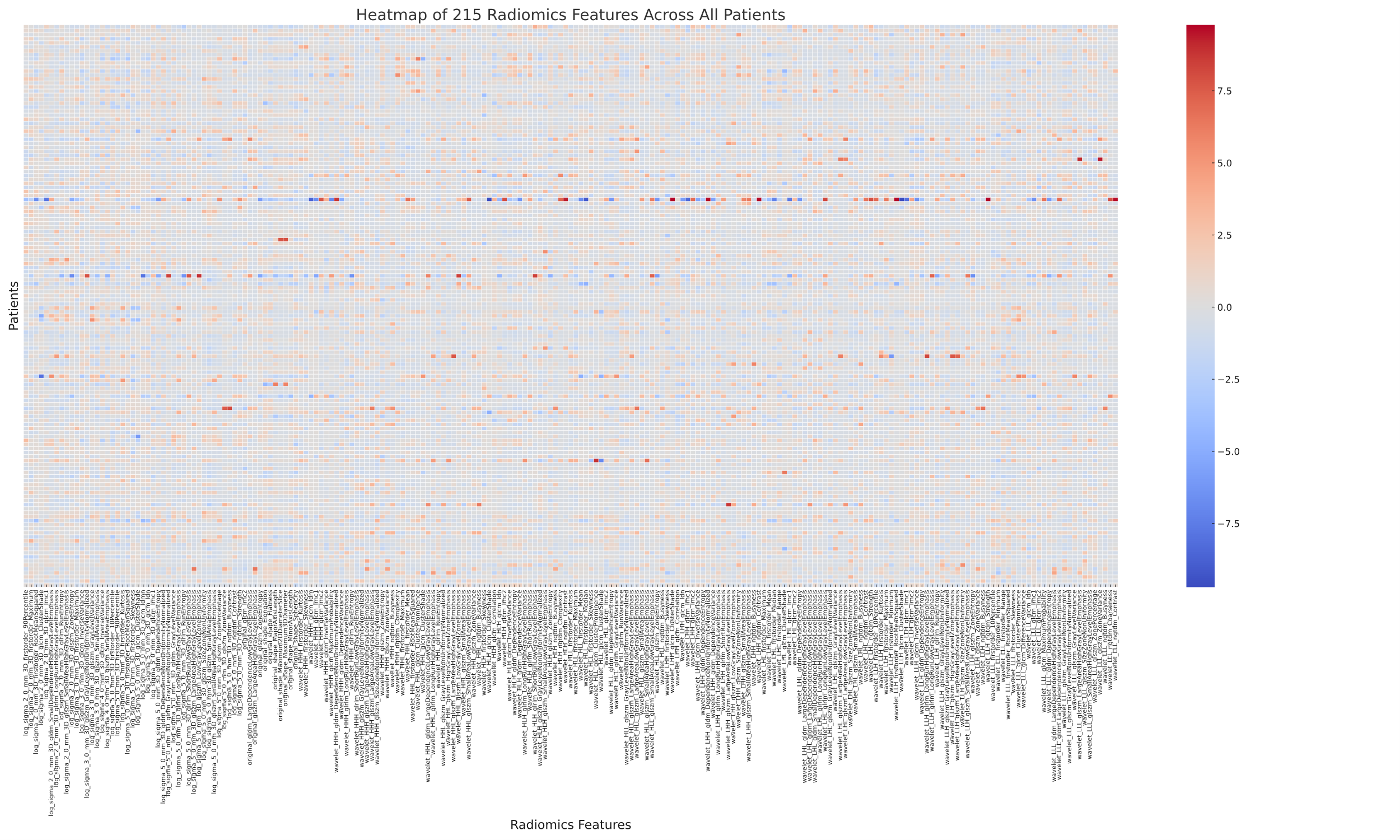


**Figure 10** The heatmaps illustrates the distribution of 215 radiomics features across all ONFH and OA cases. These features were retained following Pearson correlation analysis, where features with a correlation coefficient >0.9 were filtered to ensure non-redundancy and enhance feature uniqueness. Rows represent patients, and columns represent the retained features, with the color intensity reflecting feature values (blue for low, red for high).


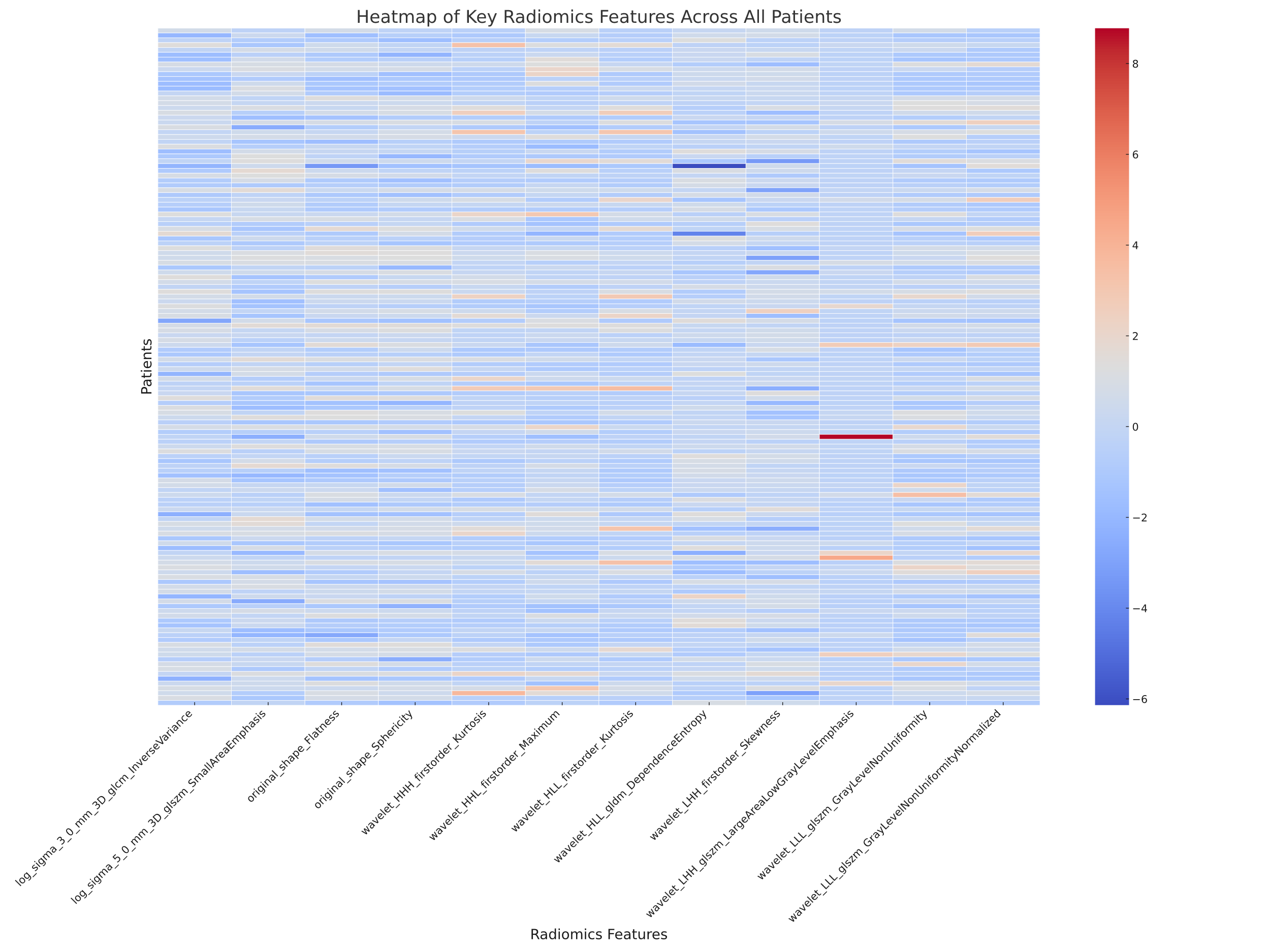
**Figure 11** This heatmap illustrates the 12 key radiomics features identified using LASSO regression to refine the feature set and enhance model performance. LASSO applied regularization by shrinking coefficients of insignificant features to zero, with the optimal λ determined through 10-fold cross-validation to minimize error. Rows represent patients, columns represent features, and the color scale reflects feature intensity (blue: low, red: high), highlighting the most relevant predictors for differentiating ONFH and OA.
